# INTEREST GROUP SESSION—RELIGION, SPIRITUALITY AND AGING: KEEPING FAITH ALIVE: ADVANCING THE NEXT GENERATION OF RELIGIOUS AND SPIRITUAL INQUIRY IN GERONTOLOGY

**DOI:** 10.1093/geroni/igz038.1302

**Published:** 2019-11-08

**Authors:** Alex J Bishop, James W Ellor

**Affiliations:** 1 Oklahoma State University, Stillwater, Oklahoma, United States; 2 Baylor University Garland School of Social Work, Texas, United States

## Abstract

A compelling body of evidence suggests that church attendance and spiritual practices increase as persons approach the end life. In fact, a growing number of baby boomers in the United States are returning to church and reconnecting with their faith communities. A primary reason for this surge in religious engagement involves a personal desire to turn away from the material pursuits of life and achieve a sense of meaning and fulfillment amid increasing frailty and vulnerability. However, most religious institutions do not have the necessary resources to support an ever increasing older membership. The graying of religion will require gerontologists to develop innovative approaches to advance theoretical thinking, measurement and assessment practices, and program evaluation. Therefore, the purpose of this symposium is to advocate and synergize a new generation of religious and spiritual inquiry in gerontology. Of particular interest is exposing theoretical models, measurement strategies, mixed-methodologies, and evidence-based findings to promote and sustain positive faith connections as persons grow old. Empirical advancements surrounding commitment and practice of one’s religious faith in old age, evolving feelings of religious belief versus doubt, disposition to seek and engage in acts of forgiveness, and faith-community responses to dementia care will be shared. Implications relevant for building interdisciplinary and transdisciplinary connections across behavioral and social science research, social work, clinical counseling, and pastoral ministry will be highlighted.

